# 

**Published:** 1894-09

**Authors:** 


					﻿Vol XIV^
SEPTEMBER, 1894.
No. 9.
THE
HOMEOPATHIC PHYSICIAN,
A Monthly Journal of Homœopathic Materia Medica
and Clinical Medicine.
“ He it a freeman whom truth maket free, and all are tlavet betide.”
“ Seek the Truth: come whence it may, cotl what it will."
, KDITZD AND PUBLISHED BY
WALTER TVL JAMES, M. D.
PHILADELPHIA :
USTo. 1231 LOCUST STREET.
LONDON:
ALFRED HEATH & CO., Bout Agknts fob Obbat Britain,
114 Ebury Street, Eaton Square.
'Yearly Subteription, $2.SO, invariably in advance. Single numberi, fS etc.
Baund at tha Philadelphia PtM-OBaa aa SaaenAOaaa Mail Matter.
				

